# Integrating preexposure prophylaxis into gynecologic care: determinants and strategies

**DOI:** 10.3389/fpubh.2026.1868869

**Published:** 2026-07-02

**Authors:** Whitney C. Irie, Eric Ganz, Lara MacIntyre, Amelia Coyle, Stephen Wagner, Douglas Krakower

**Affiliations:** 1 Bursky School of Public Health, Washington University in St. Louis, St. Louis, MO, United States; 2 Department of Obstetrics and Gynecology, Beth Israel Deaconess Medical Center, Boston, MA, United States; 3 Department of Population Medicine, Harvard Medical School and Harvard Pilgrim Health Care Institute, Boston, MA, United States; 4 The Fenway Institute, Fenway Health, Boston, MA, United States; 5 Division of Infectious Diseases, Department of Medicine, Beth Israel Deaconess Medical Center, Boston, MA, United States

**Keywords:** gynecology, HIV prevention, implementation determinants, implementation science, OB/GYN, PrEP (preexposure prophylaxis), qualitative research, women’s health

## Abstract

**Background:**

Preexposure prophylaxis (PrEP) is highly effective for HIV prevention, yet cisgender women remain underrepresented among PrEP users relative to their HIV burden in the United States. Obstetrics and gynecology (OB/GYN) settings—where many women receive routine sexual and reproductive health care—represent a promising but underutilized venue for PrEP delivery. Despite professional guidance endorsing PrEP discussion and prescribing in OB/GYN practice, implementation remains limited.

**Methods:**

We conducted a qualitative implementation study with two linked components in an academic OB/GYN clinic: semistructured interviews with 12 clinicians and clinical staff, and four multidisciplinary stakeholder workgroups focused on strategy refinement. Interview transcripts were analyzed using directed content analysis guided by the Consolidated Framework for Implementation Research (CFIR 2.0). Finalized determinants were brought to stakeholder workgroups, where participants reviewed interview findings and collaboratively identified and prioritized implementation strategies. Strategies were labeled using Expert Recommendations for Implementing Change (ERIC) terminology.

**Results:**

Five implementation determinants were identified: preventive care misfit within problem-focused visit structures, limited cognitive integration of PrEP into routine gynecologic care, fragmented care continuity and unclear role ownership, administrative and access burden, and lack of patient activation infrastructure. Stakeholders concluded that implementation failure reflected a workflow placement problem rather than a motivation problem. Strategies requiring clinicians to absorb additional counseling or coordination tasks during already time-limited visits were viewed as unlikely to succeed. Stakeholders instead refined a clinic-facing strategy bundle centered on nurse-led PrEP navigation, supported by EHR-embedded referral prompts, standardized handoff workflows, brief provider education and protocol clarification, pharmacy and insurance coordination partnerships, and structured tracking and follow-up systems.

**Conclusion:**

PrEP underimplementation in this gynecologic setting appeared to be driven less by clinician willingness than by the absence of a reliable workflow location for counseling, coordination, and follow-up. A nurse-led navigation model supported by referral prompts and access coordination may offer a feasible approach to integrating PrEP into women’s health care. Future work should test this strategy bundle in multi-site women’s health settings and pair clinic-facing approaches with patient-facing activation strategies.

## Introduction

Preexposure prophylaxis (PrEP) is a highly effective strategy for preventing HIV acquisition, with both daily oral and long-acting formulations demonstrating strong efficacy (1–3). Yet cisgender women remain underrepresented among PrEP users relative to their HIV burden in the United States, and Black women continue to bear a disproportionate share of new HIV diagnoses (4–6). Obstetrics and gynecology settings are therefore an important but underused venue for PrEP delivery because they provide routine sexual and reproductive health care, sexually transmitted infection (STI) testing and treatment, and longitudinal care for many women. The American College of Obstetricians and Gynecologists (ACOG) recommends that obstetrician-gynecologists discuss PrEP with sexually active patients and affirms that PrEP can be prescribed within obstetrics and gynecology practice (7).

Despite this clinical relevance, PrEP implementation in women’s health settings remains limited. Prior studies have identified barriers including limited visit time, uncertainty about prescribing and monitoring, incomplete workflow support, and access-related complexity (8–11). Less clear is how these barriers can be translated into feasible implementation strategies that fit gynecologic practice. We therefore aimed to identify clinician- and staff-perceived determinants of PrEP integration after STI diagnosis in an academic obstetrics and gynecology clinic and to refine pragmatic implementation strategies with multidisciplinary stakeholders.

## Methods

### Study design and setting

We conducted a qualitative study with two linked components: semistructured interviews with clinicians and clinical staff and multidisciplinary stakeholder workgroups focused on strategy refinement. Before data collection, the study team held two clinic-wide lunch-and-learn sessions on PrEP led by the obstetrics and gynecology clinic director, infectious diseases partners, and the principal investigator.

The study was conducted at the Beth Israel Deaconess Medical Center (BIDMC) obstetrics and gynecology clinic (Shapiro 8), an academic ambulatory practice serving approximately 35,000 patient visits annually across general obstetrics and gynecology, gynecologic oncology, urogynecology, and complex benign gynecology. The clinic includes 56 providers—attending physicians, nurse practitioners, residents, nurses, and support staff—and operates under a longitudinal continuity model in which attending physicians and nurse practitioners care for their own patient panels over time. The resident practice uses a rotating call structure in which patients may see different clinicians across visits, and residents serve as primary providers under attending supervision.

### Participants and recruitment

We used purposive sampling to include participants across major clinic roles and levels of training. Interview participants included 2 attending physicians, 3 resident physicians, 5 nurse practitioners, 1 registered nurse, and 1 medical assistant (N = 12). Prescribing authority was determined on the basis of professional role and credentials; 10 participants had prescribing authority and 2 did not. Only 1 participant reported ever having prescribed PrEP in the clinic. All interview participants provided informed consent.

Following interview analysis, findings were brought to four institutional review board–approved multidisciplinary workgroups convened between January and December 2025. Workgroup participants were recruited separately from interview participants through the clinic director and included nurses, physicians, residents, and medical assistants; some individuals who had completed an interview also participated in workgroups, though workgroup sessions were open to all clinic staff regardless of prior interview participation. Sustained participation was observed from clinic leadership, the chief of infectious diseases, and the on-site pharmacy director.

### Data collection

Interviews were conducted using a semistructured guide exploring experiences with STI care, perceived opportunities for PrEP prescribing, barriers and facilitators to implementation, and suggested supports for integration into routine gynecologic care (Appendix 1). The guide was structured around CFIR 2.0 domains to systematically elicit inner setting, outer setting, and individual-level determinants. Interviews were conducted via Zoom, audio-recorded with participant consent, professionally transcribed, and uploaded to Dedoose (SocioCultural Research Consultants, LLC) for data management. A brief demographic survey characterized participant role, years at clinic, credentials, prior PrEP prescribing experience, language fluency, and age.

Implementation strategy workgroups were co-facilitated by the principal investigator and site co-investigator. At each session, the research team presented a structured summary of interview-derived determinants, invited discussion of possible responses, and asked participants to evaluate candidate strategies based on feasibility, sustainability, and workflow fit. Sessions were not audio-recorded; contemporaneous field notes were taken, and session materials—including workflow sketches, EHR wish lists, and PrEP counseling scripts—were retained to support strategy refinement.

### Analytic approach

This study was grounded in a constructivist-interpretive paradigm, which holds that knowledge is co-constructed through the interactions between researchers and participants, and that meaning is shaped by context, experience, and social position rather than existing as a fixed, observer-independent reality. Interview transcripts were analyzed using directed content analysis, with CFIR 2.0 constructs serving as an *a priori* organizing framework. Two members of the research team independently coded transcripts using the CFIR 2.0 domains, then met to compare codes and reach consensus through discussion. Coded themes were reviewed and refined iteratively until consensus was achieved across all transcripts. Finalized determinants were subsequently brought to provider workgroup sessions, where participants reviewed and validated the interview findings and collaboratively identified implementation strategies suited to their clinic context. Strategy ideas generated through workgroup discussion were mapped to Expert Recommendations for Implementing Change (ERIC) labels by the research team to support standardized reporting and future intervention development (12, 13).

### Trustworthiness

To enhance credibility, two team members independently coded transcripts and resolved discrepancies by consensus. The principal investigator maintained analytic memos documenting coding decisions and theme development. Synthesized interview findings were presented back to workgroup participants for review, clarification, and refinement. The team also considered disconfirming excerpts during thematic consolidation. Researcher positionality and reflexivity are described in Appendix 2.

### Ethical approval

The Beth Israel Deaconess Medical Center Institutional Review Board approved the study (IRB #2023P000934). All participants provided informed consent. Interview participants received a $50 gift card; workgroup participants did not receive an incentive.

## Results

### Participant characteristics

Interview participants represented a range of clinical and operational roles relevant to PrEP implementation in gynecologic care, including 2 attending physicians, 3 resident physicians, 5 nurse practitioners, 1 registered nurse, and 1 medical assistant. Ten of the 12 participants had prescribing authority based on professional licensure or credentials. Only 1 participant, an attending physician, reported ever having prescribed PrEP in the clinic. Years at the clinic ranged from 2 to 24 years although baseline PrEP knowledge was not formally assessed, participant responses during interviews suggested a spectrum of familiarity: most participants had general awareness of oral PrEP, fewer were familiar with injectable formulations or current monitoring protocols, and comfort with eligibility criteria varied across role type. Notably, limited familiarity did not appear to be the primary driver of non-prescribing; participants across knowledge levels attributed their hesitation primarily to workflow and structural factors rather than uncertainty about PrEP itself. Representative interview domains and questions are presented in Table 1.

**Table 1:** Selected interview guide domains and representative questions.

CFIR 2.0 domain	Representative interview questions
Inner setting – structural characteristics	When you see a patient for a regular gynecology visit, what sexual health and prevention topics must be addressed? What are your top priorities during a typical clinic encounter?
Characteristics of individuals – knowledge and self-efficacy	How would you describe your familiarity with current PrEP guidance and monitoring recommendations? Thinking about patients seen in this clinic, do you feel PrEP is an appropriate option to suggest? Why or why not?
Inner setting – implementation climate and readiness	What resources, supports, or workflow changes would be needed to make PrEP prescribing more routine in this clinic? What would make you more or less likely to refer a patient for PrEP?
Outer setting – patient needs and access	What barriers do patients face in accessing PrEP after it is recommended? Are there insurance, pharmacy, or other access issues that have come up when discussing PrEP with patients?
Implementation process – strategy ideas	If a PrEP navigator were available in the clinic, how do you imagine their role fitting into your workflow? What would need to be in place for that model to work reliably?

Questions shown are representative examples; the full interview guide is provided in Appendix 1. CFIR, consolidated framework for implementation research.

### Implementation determinants

Semistructured interviews with 12 healthcare professionals and clinical staff identified 5 clinic-level determinants constraining PrEP counseling, prescribing, and follow-up in gynecologic care. Across themes, participants emphasized that barriers reflected a mismatch between the requirements of PrEP care and existing clinic workflow rather than resistance to PrEP itself. Representative quotes by theme are presented in Supplementary Table S1.

### Preventive care misfit within problem-focused visit structures

Participants consistently described gynecologic visits as organized around acute or problem-focused concerns, leaving limited time for preventive HIV counseling. PrEP discussions were viewed as difficult to incorporate into already constrained encounters. As one participant noted, introducing PrEP could become “a whole other thing… a whole conversation that could last another 10, 15, 20 min,” while another explained that in a 30-min problem visit, “you have to address their problem so you do not have time to do other things.” Even participants supportive of PrEP integration emphasized that “adding in another 15-min conversation about PrEP might not always be feasible.”

### Limited cognitive integration of PrEP into routine gynecologic care

Participants also described PrEP as insufficiently embedded in the *cognitive workflow* of gynecologic practice, that is, the mental scripts, habit-driven routines, and decision-making sequences that clinicians follow during a visit. Even when clinicians recognized its relevance, it was not yet part of standard visit scripts or routine checklists. One participant stated, “It’s not just part of our regular checklist of things that we talk about,” and another suggested that missed opportunities reflected “literally just… unfamiliarity from both patients and providers,” rather than the complexity of prescribing itself.

### Fragmented care continuity and unclear role ownership

Participants expressed hesitation about initiating PrEP when follow-up could not be reliably managed across visits. This concern was especially salient in the resident practice, where continuity was limited and patients might see different clinicians over time. One participant described the challenge of starting “something that you cannot adequately follow up on,” particularly “in the resident practice,” while another noted, “There’s not real continuity with our patients.” Participants also identified uncertainty about who should own the next step once PrEP eligibility was recognized. As one participant put it, “It’s not always clear whose job it is to take the next step once it comes up.” Notably, some participants began to articulate a possible solution within this theme, suggesting “one person in that nursing team be kind of the PrEP nurse… so that it does not get lost.”

### Administrative and access burden

Administrative complexity emerged as a major deterrent to prescribing and follow-up. Participants described insurance coverage, pharmacy coordination, and medication access as burdens that often arose after the initial clinical decision to prescribe. One participant summarized how easily patients and clinicians could get “bogged down” in questions such as “how much is the medication going to cost” and whether insurance would cover it. Another contrasted PrEP with contraception, noting, “You cannot just write it like birth control… you also need to make sure the medications are covered.” These comments suggested that implementation barriers persisted even when clinical interest in prescribing was present.

### Lack of patient activation infrastructure

Participants described the absence of patient-facing infrastructure that might normalize or prompt PrEP discussions. Without visible educational materials or cues in the clinic environment, PrEP was unlikely to be raised by patients themselves. One participant suggested “providing information in the waiting room… so they can have information to read and think about before we are just bringing it on them,” while another noted that such materials could help patients feel “this is a safe space” and “empower[] the patients to bring it up.”

### Determinant-to-strategy refinement

Across four multidisciplinary workgroup sessions, stakeholders reviewed the interview-derived determinants and discussed what types of clinic-based responses might be feasible, sustainable, and consistent with gynecologic workflow. These sessions were used to refine proposed implementation approaches rather than to evaluate intervention effectiveness. Stakeholders consistently concluded that strategies requiring clinicians to absorb substantial new counseling or coordination tasks during already time-limited visits would be unlikely to succeed.

Instead, workgroup discussion linked specific determinants to specific implementation needs. Preventive care misfit supported the use of EHR referral prompts and a standardized handoff process so that PrEP-related follow-up could occur outside the index encounter. Limited cognitive integration supported brief provider education and protocol clarification. Fragmented continuity and unclear role ownership supported a nurse-led navigation model with structured tracking and follow-up. Administrative and access burden supported stronger pharmacy and insurance coordination processes after referral. Together, these discussions yielded a clinic-facing strategy bundle centered on redistribution of implementation labor across roles rather than expansion of clinician tasks within the visit (Table 2).

**Table 2:** Interview-derived determinants and corresponding proposed implementation strategies for PrEP integration.

Determinant	Corresponding implementation strategy	Example action
Preventive care misfit within problem-focused visit structures	EHR-embedded referral prompts and standardized navigator handoff workflow	When STI-related risk is identified, the clinician places a brief EHR referral and transfers follow-up counseling to a navigator rather than adding a full PrEP discussion to the index visit.
Limited cognitive integration of PrEP into routine gynecologic care	Brief provider education and protocol clarification	Provide a concise clinic protocol, eligibility reminders, and brief scripts to help clinicians recognize PrEP indications and initiate referral consistently.
Fragmented care continuity and unclear role ownership	Nurse-led PrEP navigation with structured tracking and follow-up	Assign a designated team member to coordinate counseling, laboratory follow-up, outreach, and next-step ownership across visits and rotating clinicians.
Administrative and access burden	Pharmacy and insurance coordination partnerships	Develop a workflow for medication access support, including prior authorization assistance, pharmacy coordination, and troubleshooting coverage barriers after referral.

Strategies were identified through multidisciplinary stakeholder workgroups that reviewed interview-derived determinants and refined feasible clinic-based responses. These strategies were not tested for effectiveness in the present study. PrEP, preexposure prophylaxis; EHR, electronic health record; STI, sexually transmitted infection.

Lack of patient activation infrastructure remained an important determinant, but stakeholders viewed patient-facing materials and waiting-room activation strategies as a later-stage addition rather than part of the initial clinic-facing package.

## Discussion

The principal finding of this study is that PrEP underimplementation in this gynecologic setting appeared to reflect a workflow and ownership problem more than a motivation problem. Interview participants described multiple barriers to integrating PrEP into routine care, and stakeholder workgroups subsequently refined a set of proposed implementation strategies that aligned with those barriers. Importantly, these strategies were not tested in the present study; rather, they represent stakeholder-informed, clinic-specific approaches judged to have better workflow fit than strategies that relied primarily on asking clinicians to do more during already time-limited visits.

This determinant-to-strategy framing has direct clinical relevance for obstetrics and gynecology practice leaders. In our setting, preventive care misfit suggested the need to move PrEP follow-up work outside the index visit through referral prompts and handoff workflows. Limited cognitive integration suggested the need for simple educational and protocol supports rather than intensive retraining. Fragmented continuity and unclear role ownership suggested the value of a nurse-led navigation model with explicit responsibility for counseling, outreach, and follow-up. Administrative and access burden suggested the need for pharmacy and insurance coordination processes that extend beyond the prescribing decision itself. Taken together, these findings suggest that implementation planning for PrEP in women’s health settings may be more effective when focused on workflow redesign and redistribution of tasks than on clinician knowledge alone.

For clinic leaders seeking to expand HIV prevention, the key implementation question may be less whether clinicians endorse PrEP and more where PrEP-related work can reliably occur. In this study, nurse-led navigation was attractive because it addressed multiple barriers at once: it created role ownership, supported continuity across visits and rotating clinicians, and provided a mechanism for handling pharmacy and insurance tasks that frequently arise after the clinical decision to prescribe. Brief education remained useful, but as an enabling support rather than the primary intervention. This distinction may help explain why training alone often fails to translate into durable changes in preventive care delivery (14–16).

Our study extends prior work in women’s health settings by moving beyond barrier identification to show how stakeholders translated those barriers into a proposed clinic-facing implementation approach (17–20). Prior studies have described limited visit time, prescribing uncertainty, and workflow complexity as barriers to PrEP delivery (8, 9, 11). Our findings suggest that these barriers are best understood not as isolated knowledge deficits, but as linked operational determinants reflecting poor fit between preventive HIV care and problem-focused gynecologic visits. In workgroup discussions, stakeholders deprioritized approaches that lacked workflow fit and instead favored a strategy bundle centered on labor redistribution, referral infrastructure, and follow-up accountability.

These findings should be interpreted in light of several limitations. This was a single-site study in an academic obstetrics and gynecology clinic, which may limit transferability to settings with different staffing models, resources, and continuity structures. Although the sample included 12 participants across all major clinic role types, it remained purposive and modest in size. Findings were based on participant report; we did not independently assess PrEP knowledge, prescribing behavior, or implementation effectiveness. Only one participant reported previous PrEP prescribing in the clinic, which underscores the early stage of implementation in this setting but also limits inference about established PrEP workflows. Workgroup sessions were not audio-recorded and were not analyzed as a standalone verbatim qualitative dataset. Patient perspectives were not included in the present analysis, and patient activation remained an important determinant outside the scope of the initial clinic-facing strategy package. Future multi-site work should test whether relocating PrEP-related tasks across roles improves initiation and persistence in women’s health settings and should pair clinic-facing strategies with patient-facing interventions.

## Conclusion

In this gynecologic setting, barriers to PrEP implementation appeared to reflect workflow mismatch more than lack of clinician willingness. Stakeholders linked these determinants to a proposed clinic-facing strategy bundle centered on nurse-led navigation, referral support, access coordination, and structured follow-up. Future work should test whether this approach improves PrEP delivery in women’s health care.

## Data Availability

The datasets presented in this article are not readily available because it is a small dataset that could easily identify the participants, therefore it is not available for public use.
