# Supplementary material for: Integrating preexposure prophylaxis into gynecologic care: determinants and strategies
Source: Front Public Health. 2026 Jul 2;14:1868869. doi: 10.3389/fpubh.2026.1868869 (PMC13374895; doi:10.3389/fpubh.2026.1868869)
Supplement: Supplementary file 1 [file Table_1.DOCX]

**APPENDIX 1**

**Semistructured Interview Guide for Clinicians and Clinical Staff**

**Study Orientation Script**

Thank you for speaking with me today. We are interested in your perspectives on HIV prevention in gynecologic care, including how preexposure prophylaxis (PrEP) might be integrated into clinic workflow for women diagnosed with a bacterial sexually transmitted infection. We are developing a PrEP navigator model intended to facilitate rapid linkage to PrEP in the gynecology clinic.

In this proposed model, a PrEP navigator would be a nonclinical staff member who incorporates PrEP counseling into sexually transmitted infection treatment counseling, helps interested patients initiate PrEP, and links patients to follow-up care either in gynecology or through referral, depending on patient preference and clinic feasibility. Participants may also be asked to consider both oral and injectable PrEP options, recognizing that local availability may differ by setting and time.

There are no right or wrong answers. We are interested in your honest views about whether this model fits your clinic, what barriers may arise, and what would be needed to make implementation feasible.

**Opening Instructions for Interviewer**

- Introduce the study and review the consent information in accordance with the approved protocol.
- Explain that participation is voluntary and that participants may decline any question.
- Review confidentiality and audio-recording procedures.
- Answer any participant questions before beginning the interview.
- If handouts on PrEP or monitoring are used, provide them to the participant at the points indicated below.

**Section 1. Information About the Study and Care Model**

- Thank the participant for taking part in the interview.
- Explain that Black cisgender women experience substantial sexual health inequities and may be less likely to hear about PrEP from health care providers.
- Explain that the proposed intervention is a PrEP navigator model intended to support rapid linkage to PrEP for women diagnosed with a bacterial sexually transmitted infection in the gynecology clinic.
- Describe that the proposed navigator would incorporate PrEP counseling into sexually transmitted infection treatment counseling, support initiation when appropriate, and connect patients with ongoing PrEP follow-up care.
- Explain that follow-up care could occur either in gynecology or through referral to a PrEP clinic, depending on clinic workflow and patient preference.
- Explain that participants may discuss both oral and injectable PrEP during the interview.

**Section 2. Inner Setting—Structural Characteristics: Necessary Components of a Gynecology Visit**

Interviewer script: To start, I would like to learn more about the gynecology clinic and what usually happens during a typical patient visit.

- Do you discuss HIV prevention with patients? If so, how and how often?
- When you see a patient for a regular gynecology visit, what sexual health and prevention topics must be addressed?
- What are your top priorities during a typical clinic encounter?
- During a typical clinic encounter, how would you identify patients who are sexually active or who may have sexual or injection-related risk for HIV exposure?

**Section 3. Characteristics of Individuals—Knowledge, Beliefs, and Self-Efficacy**

Interviewer script: Now I would like to ask about your awareness of PrEP and your views about whether it fits gynecologic care.

- Broadly speaking, what do you know about oral PrEP for HIV prevention?
- What do you know about injectable PrEP?
- How would you describe your familiarity with current PrEP guidance and monitoring recommendations?

Interviewer instruction: Ask the participant to look at the PrEP handout if one was provided in advance. Read aloud the overview information as needed and tailor the discussion based on what the participant has already shared.

- Thinking about patients seen in this clinic, do you feel PrEP is an appropriate or not appropriate option to suggest? Why or why not?
- Thinking specifically about the clinic population that may have HIV exposure, do you feel PrEP is appropriate or not appropriate? What makes you feel that way?

Interviewer script: Let us talk more specifically about implementing PrEP care in the clinic. Please review the handout section on offering oral and injectable PrEP, and then we will discuss feasibility.

- During a typical clinic encounter, how would you identify patients who are sexually active or who may have sexual or injection-related risk for HIV exposure?
- How comfortable or uncomfortable are you initiating a conversation with Black cisgender women about their sexual health?
- What makes you feel that way?
- If comfortable, what would you say to begin the conversation?
- If uncomfortable, what do you think could increase your comfort level?
- How comfortable or uncomfortable are you initiating a conversation with Black cisgender women about PrEP for HIV prevention?
- What makes you feel that way?
- If comfortable, what would you say to begin the conversation?
- If uncomfortable, what do you think could increase your comfort level?

**Section 4. Inner Setting—Structural Characteristics: Incorporating PrEP Into Clinic Workflow**

Interviewer script: Let us move to the section on starting PrEP. Please review the sections on oral and injectable PrEP, including dosing and monitoring, and then we will discuss how this might work in your clinic.

- How complicated, if at all, would it be for every patient taking PrEP to complete recommended HIV testing and monitoring through this clinic?
- What makes you feel that way?
- If complicated, what could be done, if anything, to make it less complicated?

Interviewer script: Now let us talk specifically about prescribing PrEP.

- How prepared are you now, if at all, to prescribe PrEP to women in your clinic?
- What makes you feel that way?
- If you do not feel prepared, what would need to happen to help you feel more prepared?
- How complicated would it be, if at all, for you to prescribe PrEP to interested women?
- What makes you feel that way?
- If complicated, what could be done to make it easier?

Interviewer script: Now I would like to talk about infrastructure needs and integrating the PrEP navigator model into clinic workflow.

- How complicated would it be, if at all, for you to start a conversation about PrEP as part of a regular gynecology visit, given all your other tasks?
- What makes you feel that way?
- If complicated, what could be done to make it easier?
- How complicated would it be, if at all, for you to prescribe PrEP to interested women during the time you spend with them?
- What makes you feel that way?
- If complicated, what could be done to make it easier?
- PrEP patients often need help completing insurance or medication assistance paperwork. How, if at all, could your clinic help with that?
- Overall, what other clinic-level challenges, if any, would make it difficult to provide PrEP care at your clinic?
- What do you think could be done to address those challenges?
- Overall, how does the proposed PrEP navigator model fit with your clinic’s priorities, and in what ways might it conflict with them?
- How do you feel about patients starting PrEP in the gynecology clinic but receiving follow-up care elsewhere?
- Which follow-up approach do you prefer: continuing PrEP follow-up care in the gynecology clinic or referring patients to a PrEP clinic?
- What makes you feel that way?

**Section 5. Inner Setting—Readiness for Implementation**

- What advice do you have for developing training sessions for PrEP navigators?
- What resources do you think the clinic would need, if any, to implement a PrEP navigator model?
- In your opinion, how essential is this intervention for meeting the needs of patients who receive care in this clinic?

**Section 6. Outer Setting—Patient Needs and Resources**

Interviewer script: There is one remaining topic and then we will wrap up. The proposed PrEP navigator model encourages rapid linkage to PrEP for women diagnosed with a bacterial sexually transmitted infection in the gynecology clinic. We are particularly interested in increasing PrEP use among Black cisgender women because they are disproportionately affected by HIV compared with White women.

- What are your overall thoughts on the sexual health needs of Black cisgender women receiving care in this clinic?
- In your opinion and experience, what challenges do some Black cisgender women face in initiating PrEP?
- Why do you think those challenges exist?
- How might those challenges be addressed?
- What challenges might Black cisgender women face in obtaining PrEP medication?
- How might those challenges be addressed?
- What challenges might they face in getting follow-up PrEP care?
- Why do you think those challenges exist?
- How might those challenges be addressed?
- In what ways, if at all, would the PrEP navigator model meet the needs of Black cisgender women or address the challenges we discussed?

**Section 7. Closing**

- Do you have any final questions or comments about the proposed PrEP navigator model?
- Do you have any final thoughts about integrating PrEP into gynecologic care in this clinic?
