# Supplementary material for: Integrating preexposure prophylaxis into gynecologic care: determinants and strategies
Source: Front Public Health. 2026 Jul 2;14:1868869. doi: 10.3389/fpubh.2026.1868869 (PMC13374895; doi:10.3389/fpubh.2026.1868869)
Supplement: Supplementary file 2 [file Table_2.DOCX]

**APPENDIX 2**

**Reflexivity and positionality statement**

The qualitative data collection and analysis team included a Black woman, PhD, implementation scientist, principal investigator without a clinical role in the study clinic, and a White woman, Bachelor’s degree, research assistant with experience supporting clinical trial studies. The study team also included an obstetrics and gynecology physician co-investigator and an infectious disease physician as a mentor, both of whom are embedded in the clinical environment. The external position of the principal investigator supported independent questioning of clinic workflow norms, whereas the embedded clinicians brought contextual knowledge of local practice and feasibility. At the same time, these positions may have shaped both what participants chose to emphasize and how the team interpreted responses. Because the study team co-led clinic educational sessions on PrEP before interviews, participants may have been primed to think about PrEP implementation during the interview. To mitigate these influences, the guide was used flexibly, questions were framed in an open-ended manner, barriers and facilitators were probed with equal depth, and the team used analytic memoing and structured discussion to surface assumptions and divergent interpretations during analysis.
