# Supplementary material for: Integrating preexposure prophylaxis into gynecologic care: determinants and strategies
Source: Front Public Health. 2026 Jul 2;14:1868869. doi: 10.3389/fpubh.2026.1868869 (PMC13374895; doi:10.3389/fpubh.2026.1868869)
Supplement: Supplementary file 3 [file Table_3.DOCX]

**Appendix 3**

**Supplementary Table S1. Expanded exemplar quotes by theme**

| Theme | Exemplar quote | Professional role |
| --- | --- | --- |
| Preventive care misfit within problem-focused visit structures | “If they come in and then the provider starts to talk about it, I feel like that’s a whole other thing… a whole conversation that could last another 10, 15, 20 minutes that’s gonna add to the visit that they already have.” | Clinical Coordinator |
| Preventive care misfit within problem-focused visit structures | “The problem visits don’t, you know, are 30 minutes and you’re just meeting them and you have to address their problem so you don’t have time to do other things.” | Nurse Practitioner |
| Limited cognitive integration of PrEP into routine gynecologic care | “It’s not just part of our regular checklist of things that we talk about.” | Nurse Practitioner |
| Limited cognitive integration of PrEP into routine gynecologic care | “It’s not because it’s hard… it’s literally just because of unfamiliarity from both patients and providers.” | Attending |
| Fragmented care continuity and unclear role ownership | “All the rest of the follow-up that is required… makes it a little bit more challenging to start somebody on something that you can’t adequately follow up on, especially in the resident practice.” | Resident |
| Fragmented care continuity and unclear role ownership | “There’s not real continuity with our patients… you see any of the patients in the resident practice at any time.” | Resident |
| Fragmented care continuity and unclear role ownership | “Having one person in that nursing team be kind of the PrEP nurse… so that it doesn’t get lost.” | Nurse Practitioner |
| Administrative and access burden | “All those steps like figuring out how that’s best operationalized… how much is the medication going to cost, does my insurance cover the medication… all of that is stuff that people could get bogged down with.” | Nurse Practitioner |
| Administrative and access burden | “You can’t just write it like birth control… you also need to make sure the medications are covered.” | Nurse Practitioner |
| Lack of patient activation infrastructure | “Providing information in the waiting room… so they can have information to read and think about before we’re just bringing it on them.”  “I guess it might be helpful if patients got a little handout while they're in the waiting room just to say, heads up, you know, we're going to, we're, we, we may offer this to you or we'd like to offer this to you. So please look it over and let us know if you have any questions.” | Resident and Nurse Practitioner |
| Lack of patient activation infrastructure | “It kind of empowers the patients to bring it up and knowing that this is a safe space.” | Resident |
