# Supplementary material for: Integrating preexposure prophylaxis into gynecologic care: determinants and strategies
Source: Front Public Health. 2026 Jul 2;14:1868869. doi: 10.3389/fpubh.2026.1868869 (PMC13374895; doi:10.3389/fpubh.2026.1868869)
Supplement: Supplementary file 4 [file Table_4.DOCX]

**Standards for Reporting Qualitative Research (SRQR) Checklist**

**Manuscript: Integrating Preexposure Prophylaxis Into Gynecologic Care: Determinants and Strategies**

Appendix key: Appendix 1 = Semistructured Interview Guide; Appendix 2 = Reflexivity and Positionality Statement; Appendix 3 = Supplementary Table S1 (expanded exemplar quotes by theme).

Reference standard: O'Brien BC, Harris IB, Beckman TJ, Reed DA, Cook DA. Standards for Reporting Qualitative Research: A Synthesis of Recommendations. Academic Medicine. 2014;89(9):1245-1251.

| **No.** | **SRQR item** | **Description** | **Reported in manuscript / appendix** |
| --- | --- | --- | --- |
| S1 | Title | Concise description of the nature and topic of the study; identifies the study as qualitative or indicates the approach used. | Title page |
| S2 | Abstract | Structured summary including background, purpose, methods, results, and conclusions. | Structured abstract |
| S3 | Problem formulation | Description of the problem or phenomenon studied and its significance; review of relevant theory and empirical work. | Introduction, paragraphs 1-2 |
| S4 | Purpose or research question | Purpose of the study and specific objectives or questions. | Introduction, final sentence of paragraph 2 |
| S5 | Qualitative approach and research paradigm | Qualitative approach, guiding theory if appropriate, and research paradigm with rationale. | Methods: Analytic Approach; Appendix 2 |
| S6 | Researcher characteristics and reflexivity | Researcher attributes, qualifications, relationships with participants, assumptions, and management of potential influence on the research. | Appendix 2; Methods: Trustworthiness |
| S7 | Context | Setting or site and salient contextual factors. | Methods: Study Design and Setting |
| S8 | Sampling strategy | How and why participants, documents, or events were selected; criteria for adequacy. | Methods: Participants and Recruitment; Table 1 |
| S9 | Ethical issues pertaining to human subjects | Documentation of ethics approval and participant protections. | Methods: Ethical Approval |
| S10 | Data collection methods | Types of data collected; details of data collection procedures including timing and iterative processes. | Methods: Data Collection |
| S11 | Data collection instruments and technologies | Description of guides, protocols, recording, transcription, software, and related tools. | Methods: Data Collection; Appendix 1 |
| S12 | Units of study | Number and relevant characteristics of participants or sources included in the study. | Methods: Participants and Recruitment; Results: Participant Characteristics; Table 1 |
| S13 | Data processing | Transcription, data management, coding, anonymization, and data organization. | Methods: Data Collection; Methods: Analytic Approach |
| S14 | Data analysis | Process by which themes or inferences were developed, including coders and analytic procedures. | Methods: Analytic Approach |
| S15 | Techniques to enhance trustworthiness | Strategies to enhance credibility and dependability and rationale for their use. | Methods: Trustworthiness; Appendix 2 |
| S16 | Synthesis and interpretation | Main findings, interpretations, and themes or theory developed from the data. | Results; Discussion |
| S17 | Links to empirical data | Use of quotations, field-note material, or other evidence to substantiate findings. | Results; Table 2; Appendix 3 (Supplementary Table S1) |
| S18 | Integration with prior work, implications, transferability, and contribution(s) to the field | How findings connect with prior scholarship, practical implications, scope, and contribution. | Discussion |
| S19 | Limitations | Trustworthiness and limitations of the findings. | Discussion, limitations paragraph |
| S20 | Conflicts of interest | Potential sources of influence or perceived influence on study conduct and conclusions; how managed. | Title page: Conflict of Interest Disclosure |
| S21 | Funding | Sources of funding and other support and role of funders in the research. | Title page: Funding |
